# Assessing data imbalance correction methods and gaze entropy for collision prediction

**DOI:** 10.1371/journal.pone.0336777

**Published:** 2025-11-19

**Authors:** Courtney M. Goodridge, Rafael C. Gonçalves, Amélie Reher, Jonny Kuo, Michael G. Lenné, Natasha Merat

**Affiliations:** 1 Institute for Transport Studies, University of Leeds, Leeds, United Kingdom; 2 Federal Highway and Transport Research Institute, Bergisch Gladbach, Germany; 3 Seeing Machines, Melbourne, Australia; University of Lagos Faculty of Engineering, NIGERIA

## Abstract

Driver Readiness (DR) refers to the likelihood of drivers successfully recovering control from automated driving and is correlated with collision avoidance. When designing Driver Monitoring Systems (DMS) it is useful to understand how driver states and DR interact, through predictive modelling of collision probability. However, collisions are rare and generate imbalanced datasets. Whilst rebalancing can improve model stability, reliability of correction methods remains untested in automotive research. Furthermore, it is not yet clear the extent to which certain features of driver state are associated with the probability of a collision during critical scenarios. The current study therefore had two general aims. The first was to examine statistical model reliability when using imbalance-corrected datasets; the second was to investigate the predictive utility of gaze entropy and pupil diameter in assessing collision risk during critical transitions of control from a simulated hands-off SAE L2 driving experiment. Dataset rebalancing reduced prediction accuracy and overestimated collision probabilities, aligning with prior findings on its limitations. Erratic, spatially distributed gaze fixations were associated with higher collision probability, whilst increased mental workload (indexed via mean pupil diameter) had minimal impacts. We discuss why in many situations researchers should be wary of rebalancing their datasets, and underscore gaze behaviour’s importance in DR estimation and the challenges of dataset rebalancing for predictive DR modelling.

## 1 Introduction

### 1.1 Driver readiness

Driver Monitoring Systems (DMS) are a method for determining driver engagement, facilitating safe and effective transitions of control from automated to manual driving [1]. DMS is often implemented through vehicle-based systems (i.e., sensors on the steering wheel detecting the hands) or through camera-based technologies (i.e., detecting head and eye position) to infer the internal state of the driver. DMS are effectively trying to detect whether a driver is “ready” for a potential transition of control; however, what constitutes a “ready” driver remains an open question.

Driver Readiness (DR) (also known as driver availability, [2]) has been defined as the likelihood of drivers successfully recovering control from the automated driving task [ISO/DTS 5283−1, 3]. In essence, DR refers to understanding whether a driver can respond quickly and safely when requested to do so, in response to a likely critical event, across a range of road scenarios. DR has therefore been proposed as a variable with temporal dynamics that shift, based upon the resources available to a driver following resumption of control [4,5]. Whilst there have been many attempts to establish standardised metrics for estimating DR [6,5,4,7] there is yet to be a consensus. This is because DR is an abstract concept that represents cognitive resources (i.e., those available for attending to and processing information) and motoric resources (i.e., being available to physically control the vehicle) alongside individual-specific factors [6]. DR cannot be directly measured; however, it is operationalised via proxy constructs that that *can* be measured, alongside being associated with a driver’s ability to resume control. These constructs include mental workload (MWL) ([8,9], Situation Awareness (SA) [10,11,12] and/or visual attention [5,13]. DR can also be defined by assessing the probability that drivers avoid an accident for a given event (i.e., the controllability of a scenario) [7], with DR being high when a scenario has a high level of controllability (e.g., a 99% chance of collision avoidance). [6] proposed a conceptual model of readiness estimation based on evidence accumulation [14] and the concept of controllability [15]. The proof-of-concept model indicated that DR could be understood as an accumulation of cognitive and motoric resources and was correlated with collision probability. This result indicates that models can define readiness thresholds using experimental data without the reliance on subjective assessments of ground truth readiness.

### 1.2 Imbalanced datasets

A limitation of analysing collisions when investigating DR is that collisions are rare occurrences and result in imbalanced datasets. Imbalanced data is when one data class (i.e., the minority class) contains fewer samples than another class (i.e., the majority class). This can result in reduced accuracy for identifying infrequent cases through modelling, as they are more likely to be predicted as very rare occurrences [16,17]. However, this can be problematic for a range of disciplines, including medical diagnoses [18,19], fraud detection [20], natural disaster forecasting [21], and biological anomalies [22], because the minority class contains vital information. For DR, this is a clear weakness given that it has been assessed and defined through scenario controllability [15].

A key question is how this imbalance can be solved to ensure accurate modelling of minority classes. A common approach is data re-sampling. Three re-sampling methods are often proposed: under-sampling involves creating a subset of the original data by eliminating majority class instances; over-sampling involves replicating new minority classes; and some hybrid approaches exist that combine the two [23,24,25]. However, these methods have limitations. Because under-sampling eliminates instances of the majority class, highly imbalanced dataset ratios can result in a lack of data [16]. For over-sampling, there is the potential for overfitting, since duplications of the minority classes does not provide additional information from the underrepresented classes.

Synthetic Minority Over-Sampling Technique (SMOTE) has several advantages over traditional re-sampling methods. Whilst there are many variations of SMOTE [see 26] or [27] for a review), the general approach is that SMOTE creates new samples by interpolating existing data points based upon a k-nearest neighbours approach [28]. Members of the minority class are randomly selected alongside their k-nearest neighbour. The algorithm then creates a new synthetic data point by interpolating between the samples. Not only does this help capture the underlying characteristics of the minority class, but it also reduces overfitting, because the synthetic data points are not identical to the original data points. This approach is increasingly used in real world collision data [29] and simulator-based studies [30]. However, these studies have focused on predicting the fatality rate of collisions based on some pre-selected features (i.e., traffic information, weather data). A similar approach could be used to predict whether a collision is likely to happen in the first place (i.e., during a critical scenario), using ocular and psychophysiological measures that precede such an event. SMOTE could help create more reliable models of features that predict collision probability in the pursuit of understanding features that predict DR.

Despite the advantages of SMOTE, some researchers have found imbalance corrections do not result in better prediction models. [31] found that logistic regression models fitted with SMOTE-rebalanced clinical data resulted in mis-calibrated models (i.e., models that under/overestimated the probability of an outcome). Similarly, [32] conducted a simulation study and found that in all simulation scenarios, models developed without imbalance correction were consistently equal to or more reliable than models developed with imbalance corrected data. This issue is relevant for DR estimation given that collisions in experimental data tend be rarer than the prevalence of some clinical conditions; malignancy of diagnoses was 20% in the dataset used by [31]. Hence it is important to understand whether these rebalancing techniques are reliable for smaller and more imbalanced datasets.

### 1.3 Features for predicting collisions

SMOTE relies upon continuous features to simulate new data; ocular and psychophysiological measurements during automated driving are therefore candidate features for predicting collisions during proceeding transitions of control. Visual attention metrics are one feature that might warrant further investigation. [33] found that drivers who collided produced erratic fixation patterns. Whilst this was a useful study for indicating that visual attention could be used to investigate collision risk, focusing on raw fixations might be considered a superficial analysis of driver visual attention. More recent work has sought to apply Information Theory to driver fixations to assess visual scanning [34,35,36]. The application of Shannon’s entropy equation [37] has been used in a range of fields where there is high visuospatial demand [38,39,40]. Stationary gaze entropy $\left({\text{H}}_{\text{s}}\right)$ refers to the average level of uncertainty in the spatial distribution of a sequence of fixations [41]. The calculation of ${\text{H}}_{\text{s}}$ relies upon sorting fixations into spatial bins to generate probability distributions [41]. ${\text{H}}_{\text{s}}$ therefore, quantifies the predictability of fixation locations within the visual field. Because ${\text{H}}_{\text{s}}$ does not consider fixations *transitioning* from one bin to another, ${\text{H}}_{\text{s}}$ effectively refers to the level of gaze dispersion during a given period, with higher ${\text{H}}_{\text{s}}$ being indicative of a wider dispersion of fixation in the visual field [39]. *Gaze transition entropy* $\left({\text{H}}_{\text{t}}\right)$ builds on this through the application of the conditional entropy equation to Markov Chains [42]. Because ${\text{H}}_{\text{t}}$ utilises the stationary probability of gaze fixations, alongside the probability of transitioning from one state space to another, ${\text{H}}_{\text{t}}$ provides a measure of the predictability and complexity of visual scanning. Higher ${\text{H}}_{\text{t}}$ is indicative of less structured, more random, scanning patterns [41].

The application of entropy equations reflects the predictive nature of eye movements; a balancing of the top-down (expectation driven) and bottom-up (stimulus saliency driven) inputs that facilitate gaze control [43,44]. Whilst this dichotomy is too simplistic to fully characterise attentional control [45], theoretical developments such as predictive coding and active inference have re-conceptualised this “pseudo-dichotomy” and extend its association to gaze control [41]. These theories propose that the brain is always trying to minimise the error between its own predictions and the sensory feedback it receives [46,47]. Active inference extends this proposal further, suggesting that motor action is a method of reducing prediction error through selective sampling, i.e., via eye movements [48,47]. Given that gaze entropy encapsulates this predictive process, it has been proposed that these metrics can be used to infer the internal states of humans, and the effects on their objective behaviour, when operating in dynamic environments. For example. [6] successfully used gaze entropy in their mechanistic model of DR; [36] found that ${\text{H}}_{\text{s}}$ and ${\text{H}}_{\text{t}}$ predicted lane departures in sleep deprived drivers; [49] used gaze entropy to identify high task load in fighter pilots. There is also evidence that reductions in gaze entropy are associated with increased MWL during manual driving [50,40] and varying levels of automation [51,34].

Pupil diameter has been used as an indicator of mental effort and information processing in a range of clinical [52] and driving [53,54] scenarios. Whilst there are challenges associating increased pupil diameter with higher mental effort versus ambient lighting conditions [55], there is strong evidence that pupil diameter increases as a function of task difficulty [56–59], including MWL. However, there is still some debate regarding the impact of MWL on takeover safety, and especially collision risk. MWL has been associated with SA, especially in safety critical situations [60]. Whilst there is some evidence that high MWL results in increased response times and reduced takeover performance [61], this evidence comes from visual-cognitive non-driving related tasks (NDRTs), where drivers’ eyes are taken away from the road environment. It is therefore difficult to disentangle the visual and cognitive elements of this load. More generally, pure MWL tasks (i.e., tasks that only require the engagement of cognitive resources rather than the hands and eyes) do not tend to negatively impact takeover times [8,62,63,64,65]. Recent work using Convolutional Neural Networks (CNNs) has found that MWL is useful for predicting takeover safety, as measured by minimum time to collision, takeover time, and lane keeping [66]. Therefore, Lui et al [66] suggested that MWL should be incorporated into future takeover performance prediction, to aid in-vehicle monitoring systems. Given this finding, the use of pupillometry for predicting collision probability during engagement in a highly loading task warrants further investigation.

### 1.4 Current study

This study has two main aims. The first is to investigate the efficacy of rebalancing techniques in the context of collision data. Unless specifically designed for, collisions are rare in automotive experimental studies; much rarer than the 20% malignancy observed in clinical datasets [31]. And yet, testing the reliability of data imbalance methodologies on DR datasets remains largely unexplored. Hence an important aspect of the current investigation, therefore, is our aim of replicating previous analyses [31] on smaller, more imbalanced, datasets to test the performance of statistical modelling using uncorrected, under sampled, over sampled, and SMOTE-rebalanced data from a hands-off SAE L2 driving simulator experiment. A second aim of this study was to further investigate whether ocular and psychophysiological measures obtained during driving predicted the probability of a collision during critical scenarios. Given the range of features that modern DMS are able to capture, it is important to explore which ones are associated with collision probability, and to what extent.

## 2 Method

### 2.1 Participants

41 participants took part in the original study, however three were removed because they did not follow experimental instructions and eye tracking was not properly recorded. The remaining 38 participants (16 females, 22 males, mean age = 38.81 years, range = 22–65) all had normal or corrected to normal vision, and a valid UK driving license (mean number of years = 17.84, range = 4–43). All were regular drivers (mean annual kilometres = 15052.61, range = 8045–32180).

### 2.2 Apparatus and materials

**Fig 1:**
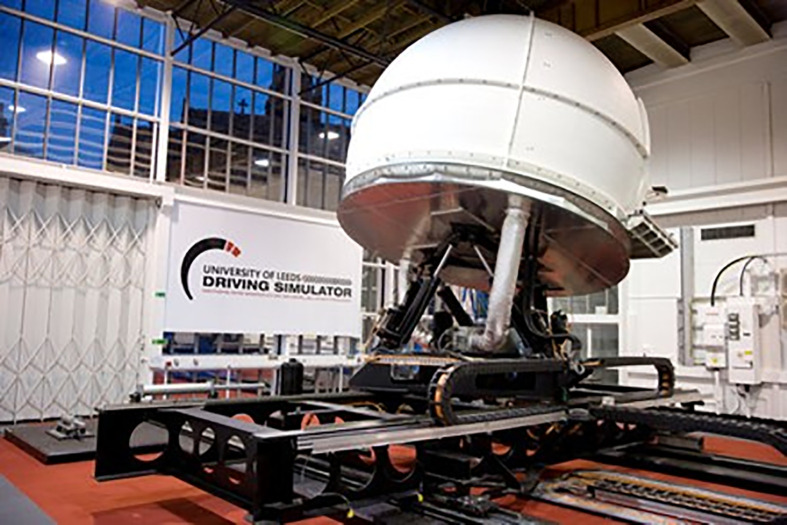
The University of Leeds Driving Simulator.

The experiment was conducted at the University of Leeds Driving Simulator; a motion-based simulator consisting of a Jaguar S-type cab within a spherical dome (see Fig 1). The dome has a 300° field of view to render the driving environment. Longitudinal and lateral movement was provided by a hexapod base and 5 m x 5 m X-Y table. All driver controls were fully operational; pedals and steering provided haptic feedback to participants. Gaze data were collected using a Seeing Machines Driving Monitoring system eye tracker sampling at 60 Hz.

### 2.3 Design and procedure

A 2 x 2 repeated measures design was used in this experiment; the two within-participants factors were MWL and takeover criticality. MWL was manipulated over two levels: a no-load condition, where drivers only monitored the road environment during hands-off SAE Level 2 driving, or a high load condition, whereby drivers had to complete a 2-back task during the hands-off SAE Level 2 driving. The criticality of the event was operationalised by manipulating the time budget of a rear-end scenario. Large time budgets were categorised as less severe (i.e., a time to collision (TTC) = 5 s) as they allowed the driver to successfully take over in most critical events; smaller time budgets (TTC = 3 s) produced critical events that could have resulted in collisions if drivers did not take over in time. These specific values were chosen as previous research has demonstrated that a 3 s TTC produces highly critical situations, whereas 5 s TTCs allow drivers sufficient time to take over [67,68,33].

**Fig 2:**
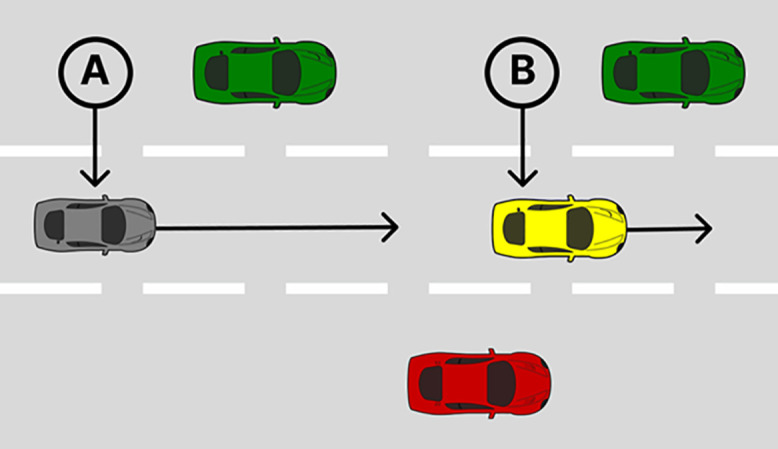
Schematic representation of an event, adapted from [34]. The ego vehicle (A) and lead vehicle (B) travelled on a three-lane motorway. The lead vehicle matches the ego vehicles speed at 25 m. After approximately 2 minutes of using the hands-off SAE L2 driving system, the lead vehicle decelerates at 5.55 ${\text{m}/\text{s}}^{\text{2}}$ (TTC = 3 s) or 2 ${\text{m}/\text{s}}^{\text{2}}$ (TTC = 5 s) for critical trials. For non-critical trials, a RTI was delivered but the lead vehicle did not decelerate.

Informed consent was obtained, both written (in terms of signing a consent form) and verbally (by asking the participant if they consented to take part in the research), and standardised instructions were delivered. All procedures were approved by the University of Leeds Research Ethics Committee (Reference code: 2022-0353-206). Participants were recruited for this study between 27/09/2024–28/10/24. The experiment was comprised of two experimental drives on a three-lane motorway. During one drive participants completed 2-back and during the other they did not; for both drives their priority was to monitor the road environment for potential hazards. At the beginning of the experimental drive, participants initially drove in the middle lane for 30 s and maintained a speed of 70 mph (112.65 kph). Following this, the hands-off SAE L2 driving system was engaged. After approximately two minutes of using the hands-off SAE L2 driving system, a request to intervene (RTI) was delivered via a short auditory tone. Simultaneously, a steering wheel icon on the human-dash-based machine interface (HMI) changed from steady green (automation engaged) to flashing red (intervention required). Once the transition of control had been completed, participants drove manually for approximately 30 s, after which the hands-off SAE L2 system reengaged. There were 10 events; four were critical (two at TTCs = 3 s and two as TTCs = 5 s), with the deceleration of the vehicle being triggered as soon as the RTI was delivered. The remaining six were non-critical; two without a lead vehicle and four with a lead vehicle that did not decelerate. These were included to mitigate learning effects. Ambient traffic flowed in the left and right lanes to provide sufficient bottom-up sensory input to facilitate driver scanning (Fig 2).

## 3 Analysis

Trial averaged data, analysis code, and models can be found in the following link: https://osf.io/nawg2/.

### 3.1 Data preparation

**Fig 3:**
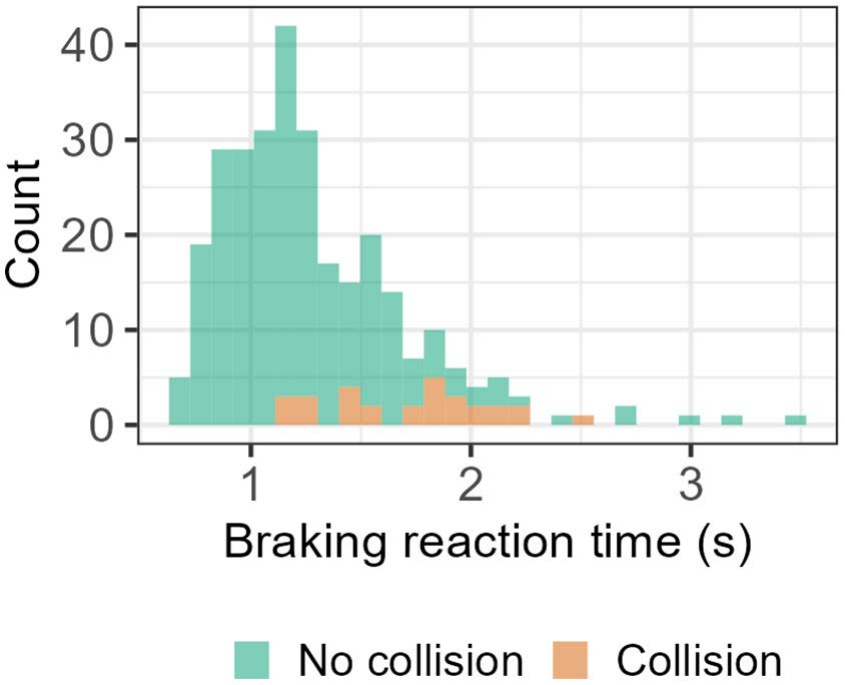
Distribution of braking reaction times during collision and no collision trials.

${\text{H}}_{\text{s}}$ and ${\text{H}}_{\text{t}}$ were calculated using fixation data from the two-minute hands-off SAE L2 automation period. It was then normalised by dividing by the maximum entropy and transformed into z-scores to improve model parameter interpretability. For the pupillometry data, blinks were removed via linear interpolation and a mean pupil size was computed by averaging left and right pupil diameter. The data were filtered with a low pass butterworth filter for smoothing. Mean pupil diameter was then calculated for the period where drivers were using the hands-off SAE L2 driving system, with the values converted to z-scores. There were 294 trials in total, of which 29 were collisions: a prevalence of 10%. Braking reaction times tended to be slower for collisions (M = 1.713 s, SD = 0.365 s) relative to non-collision trials (M = 1.238 s, SD = 0.407 s) (see Fig 3). All collisions occurred with the TTC = 3 s criticality but were approximately equally distributed between no load (16) and high load (13) conditions.

### 3.2 Imbalance correction

The SMOTE algorithm randomly selected members of the minority class (i.e., a collision) along with their k-nearest neighbour. New samples were created by interpolating between the selected sample and one of its neighbours. Interpolation involved calculating the feature vector difference between the minority sample and one of its neighbours and multiplying the difference by a random value between 0 and 1 [28]. This process can be represented as follows:


$${\text{x}}_{\text{new}}=\;{\text{x}}_{\text{i}}+\;\lambda *\left({\text{x}}_{\text{zi}}-\;{\text{x}}_{\text{i}}\right)$$ (1)

**Fig 4:**
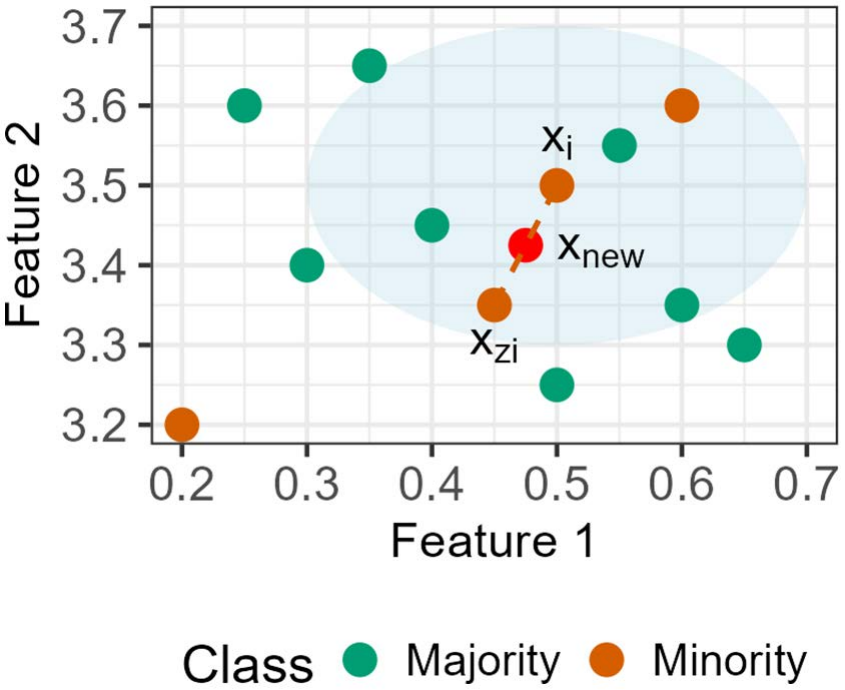
SMOTE visualisation. Shaded ellipse denotes the k-nearest neighbours (in this example, 3). A random sample of the minority class (i.e., ${\text{x}}_{\text{i}}$) and one of its nearest neighbours (i.e., ${\text{x}}_{\text{zi}}$) is selected. A new data point ${\text{x}}_{\text{new}}$ is computed on the line between the two.

Where λ is a random number in the range of [0, 1]. This creates a sample on the line between ${\text{x}}_{\text{i}}$ and ${\text{x}}_{\text{zi}}$ (see Fig 4).

**Fig 5:**
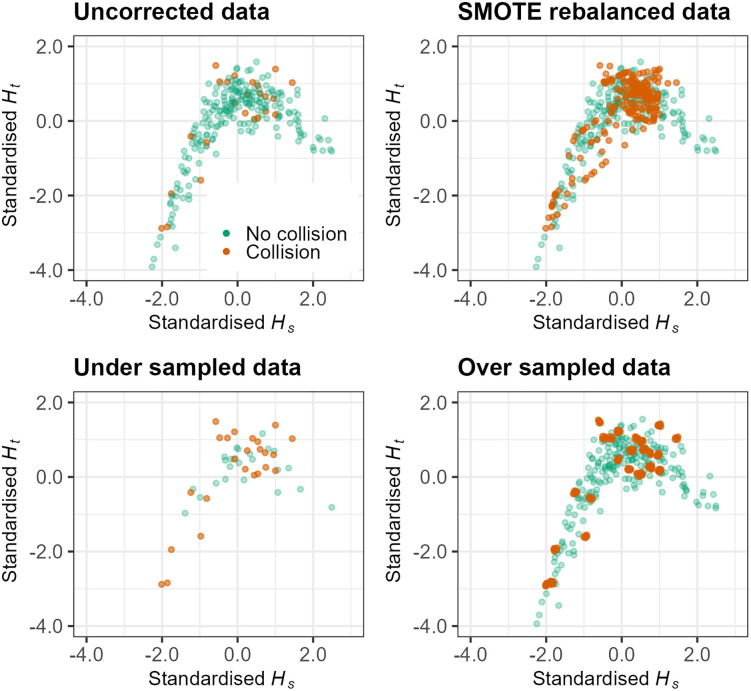
Visualisation of the uncorrected and rebalanced data. Orange data points represent collisions (i.e., the minority class) whereas green data points represent transitions of control where there was no collision (i.e., the majority class).

${\text{H}}_{\text{s}},\;{\text{H}}_{\text{t}}$, and mean pupil diameter were used as the features and the k-nearest neighbours was set at k = 5. For under sampling, the majority class was reduced by removing random cases until the majority and minority class were the same size. For over sampling, the minority class was increased by resampling cases from the minority class until the minority class was the same size as the majority class; this results in an artificially balanced dataset containing duplicate minority class cases. Fig 5 visualises the uncorrected and rebalanced datasets in terms of collisions and no collisions during transitions of control, as a function of ${\text{H}}_{\text{s}}$ and ${\text{H}}_{\text{t}}$.

### 3.3 Statistical modelling

#### 3.3.1 Overfitting and penalised logistic regression.

When using statistical models to investigate predictive markers of an outcome (i.e., the probability of having a collision during a transition of control), accuracy and generalisability are key model diagnostics. Accuracy refers to being able to predict an outcome; generalisability refers to being able to predict an outcome given new data [69]. Model fitting is a critical concept when attempting to enhance model accuracy and generalisability, with *overfitting* being of particular concern; this is where the analysis fits to the sample-specific variations rather than general underlying relationships [70]. Whilst an overfitted model may be accurate on the dataset it was developed on, the model may not generalise well to new data. Data splitting into fitting and testing datasets is one way of reducing overfitting given the rarity of having two separate datasets [71,69].

Overfitting is also relevant to imbalance correction techniques. There is a general assumption that smaller dataset sizes are prone to overfitting [72]; hence imbalance correction techniques, such as under sampling, may be particularly poor for the generalisability of prediction models. Conversely, the generation of synthetic data derived from SMOTE is thought to add variation to the minority class and thus combat overfitting [31]. Another method of reducing overfitting is the regularisation process implicated in penalised regressions. Penalised regressions impose penalties on the model for having coefficients that do not contribute to towards predicting the outcome variable [71], with regularisation being the method of shrinking unimportant coefficients down to zero. This helps with overfitting because reducing the estimated coefficients makes the model less sensitive to the structure of the fitting dataset, thus increasing the generalisability [73,71]. There are three general methods of regularisation when using penalised regressions. The Least Absolute Shrinkage and Selector Operator (LASSO) [74] shrinks coefficients variables that do not contribute to predicting the outcomes variables to zero; adaptive LASSO [75] shrinks coefficients towards zero, but not exactly zero, resulting in all variables being included in the final model; elastic net [76] is a process that is a combination of the two, shrinking some coefficients *towards* zero and others to exactly zero. Adaptive LASSO and elastic net are effectively extensions of LASSO and incorporates what is known as the L_2_ penalty on coefficients [77]. This penalty is the same as that imposed in Ridge regression and encourages the sum of squares of the model parameters in the model to be small [78].

Previous research investigating risk prediction in imbalanced datasets have compared LASSO and adaptive LASSO regularisation procedures [71,79, 31]. Hence when comparing imbalance correction methods on collision data, the current analysis investigated the impact on both standard logistic regression models and penalised logistic regression models. Adaptive LASSO was used rather than standard LASSO because it has been suggested that L_2_ penalty regularisation is more beneficial when indicators may co-vary [80,81]. Given the covariation between the gaze entropy measures, adaptive LASSO was selected as the regularisation procedure for the penalised regression models.

#### 3.3.2 Model structure and diagnostics.

Logistic regression models were fitted to estimate the log-odds of a collision via a linear combination of an intercept $\left(\beta_{\text{0}}\right)$, ${\text{H}}_{\text{t}}\;(\beta_{H_{t}})$, ${\text{H}}_{\text{s}}\;(\beta_{H_{s}})$, mean pupil diameter $\left(\beta_{D_{m}}\right)$, and interactions between these variables. A binomial distribution with a logit link function was used to model this:


$$Y_{i}\;\sim \;Bernoulli\left(P_{i}\right)$$

$$ln\;\left(\frac{P}{\text{1}\;-\;P}\right)=\left(\beta_{\text{0}}\right)+\left(\beta_{H_{t}}H_{t}\right)+\left(\beta_{H_{s}}{\text{H}}_{\text{s}}\right)+\;\left(\beta_{D_{m}}D_{m}\right)+\left(\beta_{H_{t}:H_{s}}H_{t}H_{s}\right)+\;\left(\beta_{H_{t}:D_{m}}H_{t}D_{m}\right)+\;\left(\beta_{H_{s}:D_{m}}H_{s}D_{m}\right)+\;\left(\beta_{H_{t}:H_{s}:D_{m}}H_{t}H_{s}D_{m}\right)$$ (2)

For the penalised logistic regression, another element to consider is the strength of the penalty that shrinks the coefficient; this is denoted as the lambda parameter (λ). The λ is often selected via k-fold validation with the value of λ that minimises the binomial deviance being chosen.

To investigate model performance the dataset was randomly split into fitting and testing sets using a 4:1 ratio. The fitting set was either left uncorrected or was pre-processed using SMOTE, under sampling, or over sampling. Resulting models were applied to the test set to obtain model performance; this was assessed via classification accuracy, K-fold (K = 5) cross validation prediction error, calibration (via calibration intercepts), and an analysis of the area under the curve (AUC) of the receiver operator characteristic (ROC) [82]. Calibration refers to the reliability of the predictions; to obtain calibration intercepts, the original model was used to predict the probability of a collision for the test data set. These predicted probabilities were then compared to actual outcomes via another regression model. If *β*_0_ ≈ 0, the predictions aligned well with the outcome. If *β*_0_ < 0 (probabilities were overestimated) then the model was not well calibrated. By accuracy, we refer to the proportion of drivers correctly classified as being at high or low risk of a collision. We set the risk threshold at .10 based on a previous study that aimed to estimate collision injury and recommended thresholds for classifying collisions with severe injuries at .10 [83]. A risk threshold refers to a probability value that is used to classify instances into different categories based on a model’s predicted probabilities. To make a binary classification (e.g., collision or no collision based on the state of the driver), you need a threshold. A threshold of .10 means any instance with a predicted probability greater than or equal to .10 is classified as belonging to the positive class (e.g., a collision); anything below that is classified as the negative class (e.g., no collision). Whilst .10 might seem unusually low, a lower risk threshold is often used when minimising false negatives is prioritised. This is often in situations where the cost of a missed positive (false negative) is significantly higher than the cost of a false positive. In the current context, the risk of missing a potential collision based on driver state is far greater than the cost of falsely predicting a collision might occur, when it does not. This approach ensures that more potential positive cases are identified, even if it means incorrectly classifying some negative cases as positive. However, more research is needed on specific risk thresholds that should be implemented in future DMS given that an algorithm that is too sensitive (i.e., predicts a collision based on driver state when there is no hazard) risks reducing driver trust in the system. The AUC of the ROC indicates a model’s ability of classifying a binary outcome. It visualises the trade-off between the true positive rate (i.e., the sensitivity) and the false positive rate (i.e., 1 – specificity). The true positive rate refers to the proportion of actual positives (i.e., the proportion of collisions) whereas the false positive rate refers to the proportion of actual negatives that are incorrectly identified as positives. The AUC therefore represents the overall performance of the model with an AUC of 1 being a perfect classifier, and 0.5 being a random classifier.

Finally, the nature of the hierarchical data structure from the repeated measures design would normally necessitate a multilevel modelling approach to account for the clustering of observations. However, the model using SMOTE-rebalanced data failed to converge with random slopes or random intercepts. This is likely because when SMOTE generates new synthetic data points, each observation must be assigned their own individual identifier akin to a participant identification number. This creates a situation where approximately 200 synthetic participants are generated with only one observation per participant. As a result, the random effects structure is highly unstable precluding the fitting of random intercepts. Because it is not feasible to compare model performance of mixed-effects (uncorrected, under sampled, and over sampled models) with fixed-effects (SMOTE model) models, a decision was taken to fit only fixed effects models across all four datasets. When using a fixed effects approach to model hierarchical data, the regression estimates themselves remain unbiased. However, *the precision* of the estimates will be overestimated and thus will need adjusting [84]; this largely pertains to the confidence intervals and the p values. Therefore, the precision of the estimates was corrected using clustered standard errors [85] to allow valid interpretation of the p values and confidence intervals for model interpretation. One thing to note is that model parameter interpretation was only available for the standard logistic regression models. The regularisation involved in penalised logistic regression aims to balance the bias-variance trade-off [73]. By shrinking the coefficients (i.e., by adding bias), the model becomes less sensitive to the fitting data characteristics. This results in smaller changes in predictions when estimating the model on a testing dataset (i.e., reduced variance). Because the penalised estimation influences the variance associated with coefficients, the standard errors are not meaningfully interpretable. Hence constructs such as confidence intervals and p values do not exist for LASSO estimates [86].

## 4 Results

### 4.1 Model performance

**Fig 6:**
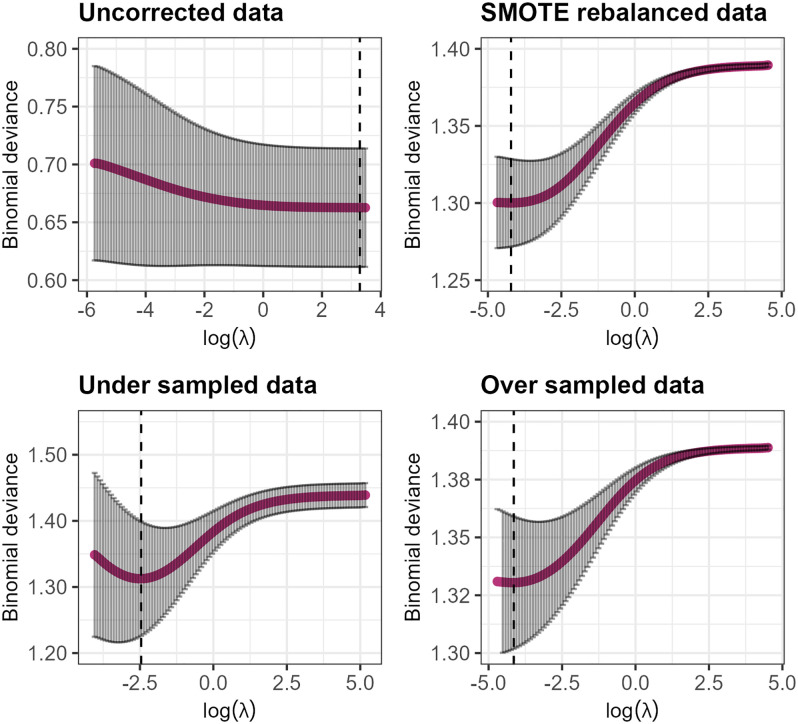
Visualisation of the hyperparameter tuning for the λ parameter. Dashed lines represent the value of log(λ) that minimises the binomial deviances for each dataset.

Visualisation of the λ hyperparameter tuning for penalised logistic regression models is highlighted in Fig 6. Following the tuning, the λ parameters that minimised the binomial deviance were incorporated into the final model and model performance was assessed.

**Fig 7:**
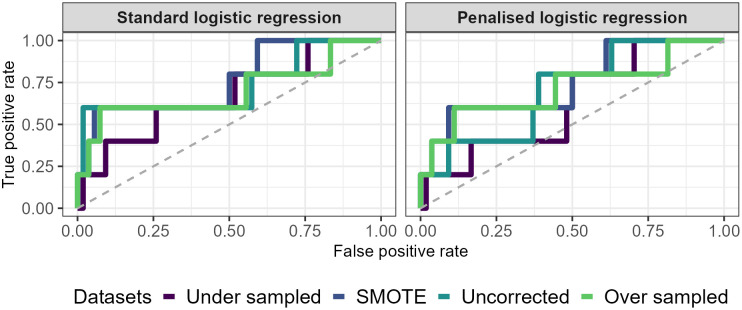
Visualisation of the AUC values for each dataset and model. It is clear that the under and oversampled models have the smaller AUC values relative to uncorrected and SMOTE-rebalanced data.

For standard and penalised logistic regression models, prediction accuracy was highest when using the uncorrected dataset relative to the SMOTE-rebalanced, under sampled, and over sampled datasets (see Table 1). However, the drop-off in accuracy was much less pronounced for penalised logistic regression relative to standard logistic regression. For example, the third worst performing penalised logistic regression model in terms of accuracy was only five percentage points off the best performing standard logistic regression model. In general, the penalised logistic regressions had much higher accuracy overall, suggesting they were better at predicting outcomes in the testing dataset. This is likely because the regularisation process of adaptive LASSO improved the generalisability of the model to new data. Analysis of the AUC revealed that it was slightly higher for SMOTE-rebalanced data when using standard and penalised logistic regression, however this was not by a considerable amount. Under and over sampled data tended to result in the worst AUC for both model types (see Fig 7).

**Table 1:** Model performance.

Model	Dataset	Accuracy	K-fold cross validation prediction error	AUC
Standard logistic regression	Uncorrected	76%	0.097	0.733
	SMOTE	9%	0.227	0.763
	Under sampled	14%	0.333	0.670
	Over sampled	9%	0.230	0.701
Penalised logistic regression	Uncorrected	91%	0.185	0.703
	SMOTE	71%	0.456	0.751
	Under sampled	61%	0.467	0.625
	Over sampled	83%	0.473	0.718

**Fig 8:**
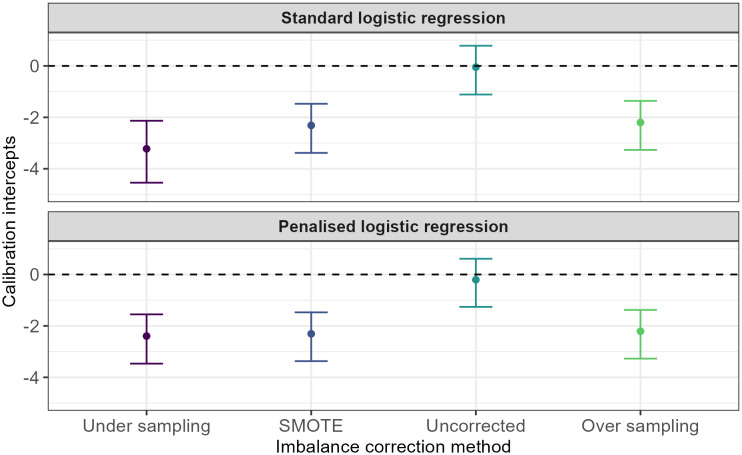
Test data set calibration for the intercepts of standard and penalised logistic regression models fitted with uncorrected, SMOTE-rebalanced, under sampled, and over sampled data.

This differences in model accuracy between uncorrected and rebalanced datasets were also encapsulated by the highly mis-calibrated intercepts that were identified in the calibration analysis (see Fig 8). For standard and penalised logistic regression models, calibration intercepts were estimated close to 0 for the uncorrected data model (standard = −0.047, [95% CI: −1.112, 0.785], penalised = −0.205, [95% CI: −1.259, 0.612]) relative to the SMOTE-resampled model (standard = −2.313, [95% CI: −3.383, −1.472], penalised = −2.306, [95% CI: −3.370, −1.473]), the under sampled model (standard = −3.223, [95% CI: −4.542, −2.133], penalised = −2.395, [95% CI: −3.467, −1.551]) and the over sampled model (standard = −2.198, [95% CI: −3.268, −1.360], penalised = −2.209, [95% CI: −3.272, −1.378]). Based on the ocular and psychophysiological data used for this study, we can assume that models fitted with imbalanced corrected datasets overestimated the probability of having collisions when driver’s gaze entropy and mean pupil diameter were at average levels.

### 4.2 Model interpretation

**Fig 9:**
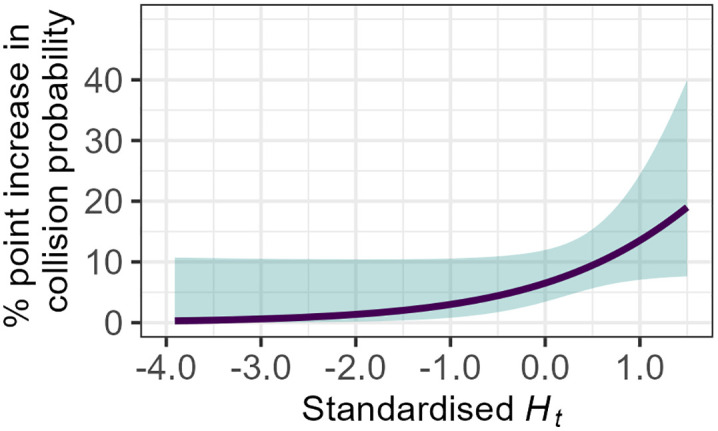
Relationship between standardised ${\text{H}}_{\text{t}}$ and the probability of a collision for average levels of ${\text{H}}_{\text{s}}$ and pupil diameter. As standardised ${\text{H}}_{\text{t}}$ increases (i.e., as transitions of gaze become more random) the probability of a collision occurring during a critical takeover increase.

Given that parameter interpretation of the penalised regression models was not possible due to the regularisation process, the standard logistic regression models were taken forward for interpretation. The model fitted with uncorrected data provided improved performance and thus was used for model interpretation. ${\text{H}}_{\text{t}}$ was predicted to significantly increase collision probability (*β*_*Ht*_ = 0.811, [95% CI: 0.079, 1.542], z = 2.173, p = 0.029) (see Table 2). For average levels of ${\text{H}}_{\text{s}}$ and pupil diameter, a one standard deviation increase in ${\text{H}}_{\text{t}}$ (~4 percentage points of normalised ${\text{H}}_{\text{t}}$) resulted in an increase in the probability of a collision by 7 percentage points. Exponentiation of the standardised coefficient $\left(e^{\beta_{H_{t}}}\;=\;\text{2}.\text{250}\right)$ revealed that this was equivalent to a medium effect size, as defined by Rosenthal [87]. This result implies that if the spatial distribution of gaze is at an average level and drivers have average levels of MWL (as indexed by mean pupil diameter), then an increase in the randomness of gaze transitions is predicted to increase the probability of a collision during critical transitions of control (see Fig 9).

**Table 2:** Model parameter estimates from uncorrected data GLM.

Predictors	Estimate	SE	z-value	p-value
$\beta_{0}$	**−2.664**	**0.339**	**−7.851**	**<0.001**
$\beta_{H_{t}}$	**0.811**	**0.373**	**2.173**	**0.029**
$\beta_{H_{s}}$	−0.064	0.267	−0.241	0.809
$\beta_{D_{m}}$	0.050	0.345	0.146	0.883
$\beta_{H_{t}:H_{s}}$	**0.517**	**0.243**	**2.123**	**0.033**
$\beta_{H_{t}:D_{m}}$	0.231	0.356	0.650	0.515
$\beta_{H_{s}:D_{m}}$	−0.063	0.255	−0.249	0.802
$\beta_{H_{t}:H_{s}:D_{m}}$	0.177	0.165	1.072	0.283

Note: N *=* 38, Observations *=* 235.

**Fig 10:**
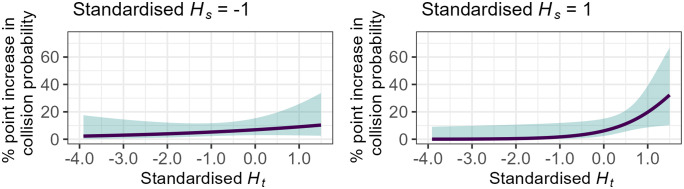
Relationship between standardised ${\text{H}}_{\text{t}}$, ${\text{H}}_{\text{s}}$, and the probability of a collision for average levels of pupil diameter. When standardised ${\text{H}}_{\text{s}}$ is lower than average (i.e., the spatial distribution of gaze is constrained), increasing standardised ${\text{H}}_{\text{t}}$ appears to have minimal effects on the probability of a collision. However, when standardised ${\text{H}}_{\text{s}}$ is over average (i.e., the spatial distribution of gaze is dispersed), an increase in the randomness of gaze transitions results in higher probability of a collision during a critical takeover.

The model also highlighted a significant interaction between ${\text{H}}_{\text{t}}$ and ${\text{H}}_{\text{s}}$ (*β*_*Ht:Hs*_ = 0.517, [95% CI: 0.039, 0.994], z = 2.123, p = 0.033). For average pupil diameter levels, increasing ${\text{H}}_{\text{s}}$ amplified the effect of ${\text{H}}_{\text{t}}$ on increasing collision probability by a further 4 percentage points. Exponentiation of the standardised coefficient $\left(e^{\beta_{H_{t}:H_{s}}}\;=\;\text{1}.\text{677}\right)$ revealed that this was also equivalent to a medium effect size, albeit smaller than the effect of ${\text{H}}_{\text{t}}$ individually. This result implies that when the spatial distribution of gaze is higher, *and* the fixations are highly random, there is a higher probability of a collision (see Fig 10).

## 5 Discussion

The aims of this study were twofold: firstly, to investigate whether logistic regression models, fitted with imbalanced corrected data, produced reliable predictions of collision probability; and secondly, to understand whether gaze entropy and pupil diameter were useful predictors of collision probability. The analysis revealed that dataset rebalancing did more harm than good. Prediction accuracy was much lower, and collision probabilities were overestimated, particularly for average levels of gaze entropy and pupil diameter. This supports previous research indicating that the application of balance correction methodologies reduces the reliability of logistic regression prediction models [31]. Modelling also revealed that penalised logistic regression models were less susceptible to the reduction in prediction accuracy, most likely due to the regularisation discarding ill-augmented parameters. Interpretation of the model parameters revealed that erratic eye movements during hands-off SAE L2 driving resulted in a higher probability of a collision during critical scenarios. When controlling for the spatial distribution of gaze and pupil diameter, every 1 standard deviation increase in the randomness of gaze transitions resulted in a 7-percentage point increase in collision probability. This effect was exacerbated when the spatial distribution of gaze was more dispersed, as highlighted by the significant interaction effect; both effects were estimated as medium standardised effect sizes.

All imbalance correction methods resulted in logistic regression models that vastly overestimated the probability of a collision for average levels of gaze entropy and pupil diameter. Whilst the models fitted in this manuscript are simplistic, they provide great impact as they demonstrate the risk of artificially rebalancing datasets when investigating DR; that DR models may be overly biased in predicting and alerting for collisions. There is already a concern that DMS have high false positive rates which can elevate the “cry wolf effect”. This effect refers to drivers not conforming to a DMS once it has already delivered a false positive [88]. This can result in drivers being less likely to adhere to future warnings once they have been shown to be unreliable. The analysis presented in this manuscript indicates that correcting data imbalances when developing collision prediction models may contribute to these false positive rates; this was evident with under and over sampling techniques, with the AUC curve frequently hitting, and sometimes surpassing, the diagonal “random classifier” reference line. Interestingly, the penalised logistic regression models appeared to reduce the negative effects of imbalance correction on model accuracy. One reason for this could be because the regularisation process resulted in models that were not overfitted to the training data and thus were more generalisable to the test dataset.

**Fig 11:**
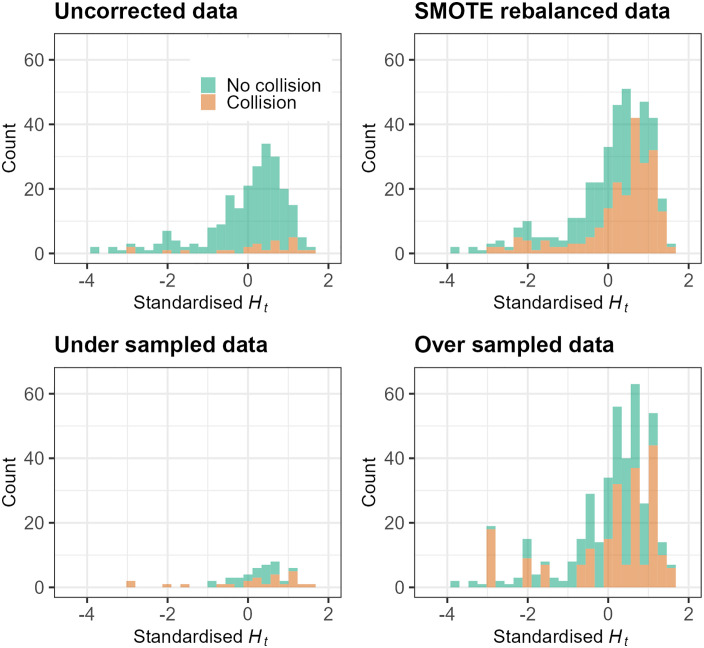
Changes in distributional structure of the sample under various data rebalancing methods.

Despite penalised logistic regression models being more accurate overall, rebalancing the datasets still significantly reduced model accuracy. One explanation for this is that rebalancing the data introduces significant bias into the sample. Regardless of the method, rebalancing merely increases or decreases the number of data points that exist in a given sample. In the case of under sampling, critical data points related to the predicted outcome may be removed and as such, may not accurately reflect the test dataset. Conversely, over sampling may unintentionally expand hidden anomalies, resulting in overfitting to training data sets. SMOTE has been proposed as a way of improving the decision boundaries between classes [89] – as may have been evidenced by the slightly higher AUC values from models fitted with SMOTE-rebalanced data – however, enhancement of *predictive accuracy* may be minor if new data contains similar properties to observations already in the dataset [90]. Another reason why data rebalancing may be ill-advised is because the data used to fit the model should ideally reflect data in the real world. By correcting the imbalance, one is inadvertently altering the distributional structure of the dataset towards something that may not be observed in the real world. This can be visualised from the current example in Fig 11. The distributional properties of ${\text{H}}_{\text{t}}$ within collision and no collision classes vary significantly; whilst uncorrected and SMOTE-rebalanced datasets still have negative skew and extended left hand tails, collisions in the extended left tail are amplified in the over sampled dataset; the distributional shape is almost non-existent in the case of under sampling resulting in significant information loss. Models should be trained on datasets whose distributions reflect the future, real-world test cases for which they will ultimately be applied; this may be a key reason not to rebalance datasets.

A final consideration when it comes to imbalanced data is that the number of minority class observations may already be sufficient. An example adapted from [91] illustrates this; sample A comprised of 1,000,000 observations with 50,000 minority classes and sample B comprised of 100 observations with 5 minority classes both have a minority prevalence of 5%. However, models fitted with sample A are likely to drastically outperform models fitted with sample B. Therefore, a researcher presented with sample B may think their problem is data imbalance, but really it is simply a problem of incomplete training data with the consequence being reduced statistical power. Given that the problem remains in this research area that collisions are rare yet important events that must be analysed to inform future DMS development, an alternative solution could be through dataset pooling from different research studies. Although datasets may vary in terms of scenario, level of automation, experimental apparatus, etc., a multilevel modelling approach can account for the variation attributed to the differing datasets. This can be implemented by assigning each dataset their own intercept in the model, with common predictors across the datasets (e.g., physiological measures, the presence of non-driving related tasks) being included as random slopes to explicitly quantify how, for example, gaze entropy predicts collision probability across different studies. A barrier to this solution is the lack of openness and transparency of automative research data. [92] found that whilst open data practices were valued by the research community, significant barriers, such as legal and organisational constraints, prevent this ethos. Support in overcoming these barriers alongside open data guidelines will be necessary to facilitate progress.

In terms of the model parameter interpretation, higher than optimal ${\text{H}}_{\text{t}}$ is indicative of top-down interference and thus related to a modulation beyond what the specific task and visual scene requires [41]. Such over-modulation is reflected by more erratic visual scanning patterns which are less efficient for the task of monitoring a driving assistance system and the road environment. In general, it has been found that impairments in visual scanning reduces hazard perception and produces higher risks of road collisions [93,94]. However, mechanistically, it is not clear how this relates to increased collision probability during critical scenarios. One suggestion that links increased ${\text{H}}_{\text{t}}$ to collision probability is hypervigilance. Increased activation in the prefrontal cortex can result in downstream effects on motor output [95]. The medial prefrontal cortex (mPFC) plays a key role in regulating emotive behaviour. It integrates information sources that influence the relative threat of a situation – i.e., an individual’s motivational state, the prior history of the stimulus history, or the similarity of the stimulus to previous threats [95,96]. This activates downstream projections to areas such as the thalamus, the amygdala, and the hypothalamus [97,98]. The result is a modulation in anxiety-related motor behaviour [95]. In clinical populations, this can result in hypervigilance due to higher expectations of, and searches for, potential threats which results in more dispersed and erratic visual scanning [99,100,101,102]. During these situations, an observer’s internal state may be over-interpreted resulting in bottom-up input being over modulated [103,104]. It is these mechanisms that are thought to contribute towards ${\text{H}}_{\text{t}}$ that is beyond an optimal range. Whilst the current sample were not clinically diagnosed, the overinterpretation of internal (top-down) states may still have manifested as hypervigilance, resulting in inefficient visual search strategies. Overall, this makes it less likely for drivers to attend towards informative cues, reducing hazard perception, and increasing the probability of collisions.

Another interesting result was that high MWL (as indexed via mean pupil diameter) did not predict collision probability during transitions of control. Previous analysis on naturalistic manual driving (Strategic Highway Research Program 2 [SHRP2] data set) has found contrasting results. [105] found that none of the 47 rear-end collisions involved engagement with a hands-free phone (i.e., an NDRT that could be considered as increasing MWL). Conversely, other analyses highlighted increased general collision risk (i.e., not just rear-end collisions) for phone conversations relative to no task references [93,106]. A more in-depth analysis of naturalistic collision data, in the context of higher levels of automation, is needed to fully understand how MWL links to collision risk. A potential starting point is the Cognitive Control Hypothesis (CCH) [107] which offers a theoretical framework for understanding how MWL may relate to collision risk. CCH proposes that MWL selectively impairs tasks that are novel and/or are inherently uncertain; such tasks rely upon cognitive control and thus compete with NDRTs for limited cognitive resources, which may result in detrimental performance if supply is not provided. Conversely, tasks that are automatised are less likely to affected by MWL given that they rely upon strong neural pathways that circumvent the executive functions necessary for cognitive control. Whilst CCH was developed for manual driving, it may be applicable to automated driving, and in particular, rear-end scenarios. Given that responding to strong looming signals (i.e., a lead vehicle braking) is likely to be automatised [62,108,109,110], it might be expected that MWL has low predictive power for collisions, under the CCH.

In the context of future DMS design, the current analysis indicates that a more dynamic approach to quantifying driver visual attention might be useful for predicting collision probability. The most recent recommendations for DMS development concentrate on visual distraction via Advanced Driver Distraction Warnings (ADDWs) or Driver Drowsiness and Distraction Warnings (DDAW). These recommendations define fixed parameters for detecting visual distractions such as single long glances (looking away from the forward road view for 3–4 s) or multiple short glances (glancing away from the forward road view for a cumulative 10 s out of a 30 s period) [111,112]. A more nuanced approach might be using a system that quantifies the optimisation of visual attention rather than relying on fixed temporal elements; gaze entropy offers one such quantification, with the results from the current study indicating that it is a feature with predictive power for collision probability. A limitation of this inference is that ocular and psychophysiological measures used in the current analysis were calculated using approximately two minutes of eye tracking data *prior* to a transition of control. This would not be a practical approach in the real world [as in 41]. Instead, future research may want to focus on methods that can dynamically identify driver visual attention and cognitive state in real time, to predict future collision potential. Another design consideration is the implementation of technology that integrates gaze entropy with the visual and driving demands of the road environment. Gaze entropy is not only modulated by the driver’s cognitive state; it also varies as a function of the complexity of the visual scene [41]. Similarly, the probability of colliding during a critical transition of control may differ according to the road environment and particular scenario. Therefore, a system that is adaptive to the environment could be more effective in predicting collision probability as it might combine driver state with the visual demands of the environment. Further research may pursue this line of investigation by understanding how the probability of colliding during a critical scenario alters dependent upon the complexity of the visual environment or road structure, to determine optimal gaze entropy ranges for specific environments.

Another limitation of the current work is that only measures of visual sampling and pupil size were used to predict collision probability. However, there are other psychophysiological signals that can be used to monitor the state of drivers in real time. [113] successfully used galvanic skin responses (GSRs) and heart rate (HR) features to predict takeover time and quality from L3 automation. [114] found that GSRs and gaze dispersion were among the top five most important features in their machine learning models for predicting takeover performance following conditionally automated (SAE L3) driving. However. HR features (e.g., standard deviation of inter-beat interval and maximum HR) were ranked least important. This corroborates with the current work in that not all psychophysiological signals provide predictive value, but that the dispersion of gaze during automated driving certainly seems to. [114] also suggested that different psychophysiological signals may be beneficial for prediction models that utilise different time windows; for example, pupillometry might be more useful when using shorter time windows because its phasic properties represent rapid changes in cognitive state that add predictive value in how drivers respond [see 115,116,117]. That is not to say that the slowly evolving tonic component of pupil size is not useful for identifying cognitive fluctuations; just that phasic responses might be more informative for understanding how drivers respond in critical situations when the engagement of cognitive resources is heavily time dependent.

Similarly, [118] found that HR and GSRs (i.e., skin conductance level) increased during takeover periods following L3 automation. Furthermore, these physiological measures could also distinguish between the secondary tasks that drivers were completing during the automated period (i.e., observing the road and system vs 1-back vs 2-back). Overall, [118] concluded that these psychophysiological indices could be used as benchmarks of takeover preparedness and performance. More recently, work by [119] found that psychophysiological signals were *indirect* predictors of takeover quality. For example, GSRs were related to takeover quality from L3 automation through the mediating variable of trust. It was suggested that drivers who trusted the automated system more would be less stressed, thus explaining the negative associations between GSRs and trust. Similarly, electrocardiograms and respiration rate were related to takeover times via the mediating variable of MWL. [119] concluded that machine learning models that use psychophysiological variables to predict takeover performance are actually estimating the psychological state of drivers. This means that additional information could be used to further improve the predictive accuracy of these models, such as traffic conditions that impact driver task load [120] and environmental factors affecting situational trust [121].

Other studies have relied upon direct measures of cognitive state for informing their prediction models. [122] found that electroencephalographic (EEG) signals were the most important features for predicting takeover quality from SAE L3 automation; specifically, power in the Alpha (8–12 Hz) and Beta (12–30 Hz) frequency bands. Whilst some driving studies have associated increased Alpha power with fatigued driving [123,124,125], others have challenged this notion and suggest that Alpha power changes may refer to fluctuations in task demands and visual input due to monotonous driving [126]. This latter suggestion supports contemporary views of top-down mediated modulations in Alpha power in the sensory cortices; it facilitates the inhibition of irrelevant input and increases responsiveness in task-relevant regions [127]. Beta power is thought to play an important role in attentional processes associated with thalamic and cortical centres of the visual system [128–135]. Research has found that the Beta frequency band is involved in facilitating the synchrony between parietal (e.g., the Lateral Intraparietal Area; LIP) and frontal cortices (e.g., the Lateral Prefrontal Cortex; LPFC) during top-down modulations of attention. Measuring neurons directly, [136] found greater synchrony between the LIP and LPFC in the Beta frequency band during a top-down visual search task; during a bottom-up “pop out” visual search LIP-LPFC synchrony was highest in the Gamma frequency band. Recent work has found similar results [137,138] which suggest top-down and bottom-up modulations of attention rely upon different frequency bands. Within the broader context of driver state, evidence that Beta power and gaze entropy both predict takeover quality suggests that takeover performance may depend on effective top-down modulation of attention. Accordingly, ${\text{H}}_{\text{t}}$ provides a reliable, non-invasive index of top-down interference, highlighting gaze control as a system of spatial prediction. Future work should investigate ${\text{H}}_{\text{t}}$ in relation to Beta activity to clarify their connection. Peripheral measures most useful for assessing drivers’ internal states are likely those closely tied to neural dynamics. Given their shared link to top-down visual attention, ${\text{H}}_{\text{t}}$ and Beta power represent a particularly important avenue for further study.

In conclusion, based upon the analysis of the current data, we can provide the following advice to researchers with imbalanced collision data: rebalancing the dataset with any method may do more harm than good in terms of predictive accuracy, model calibration, and balancing true/false positive rates. Whilst penalised regression models provide some protection from this decrease in model performance, there is no substitute for the sufficient quality and quantity of data in the first instance. Pooling similar datasets within a research domain may be a potential solution, however more research is needed to establish whether this a viable option in terms of model stability and performance.
